# Processing Non-at-Issue Meanings of Conditional Connectives: The *wenn/falls* Contrast in German

**DOI:** 10.3389/fpsyg.2021.629177

**Published:** 2021-08-10

**Authors:** Mingya Liu

**Affiliations:** Department of English and American Studies, Humboldt University of Berlin, Berlin, Germany

**Keywords:** conditional connectives, German, experiment, speaker commitment, non-at-issue meaning

## Abstract

Logical connectives in natural language pose challenges to truth-conditional semantics due to pragmatics and gradience in their meaning. This paper reports on a case study of the conditional connectives (CCs) *wenn/falls* ‘if/when, if/in case’ in German. Using distributional evidence, I argue that *wenn* and *falls* differ in lexical pragmatics: They express different degrees of speaker commitment (i.e., credence) toward the modified antecedent proposition at the non-at-issue dimension. This contrast can be modeled using the speaker commitment scale (Giannakidou and Mari, 2016), i.e., _More committed_<WENN p, FALLS p>_Less committed_. Four experiments are reported which tested the *wenn/falls* contrast, as well as the summary of an additional one from Liu (2019). Experiment 1 tested the naturalness of sentences containing the CCs (*wenn* or *falls*) and conditional antecedents with varying degrees of likelihood (very likely/likely/unlikely). The starting prediction was that *falls* might be degraded in combination with very likely and likely events in comparison to the other conditions, which was not borne out. Experiment 2 used the forced lexical choice paradigm, testing the choice between *wenn* and *falls* in the doxastic agent’s conditional thought, depending on their belief or disbelief in the antecedent. The finding was that subjects chose *falls* significantly more often than *wenn* in the disbelief-context, and vice versa in the belief-context. Experiment 3 tested the naturalness of sentences with CCs and an additional relative clause conveying the speaker’s belief or disbelief in the antecedent. An interaction was found: While in the belief-context, *wenn* was rated more natural than *falls*, the reverse pattern was found in the disbelief-context. While the results are mixed, the combination of the findings in Experiment 2, Experiment 3 and that of Experiment 4a from Liu (2019) that *falls* led to lower speaker commitment ratings than *wenn*, provide evidence for the CC scale. Experiment 4b tested the interaction between two speaker commitment scales, namely, one of connectives (including *weil* ‘because’ and *wenn/falls*) and the other of adverbs (factive vs. non-factive, Liu, 2012). While factive and non-factive adverbs were rated equally natural for the factive causal connective, non-factive adverbs were preferred over factive ones by both CCs, with no difference between *wenn* and *falls*. This is discussed together with the result in Liu (2019), where the *wenn/falls* difference occurred in the absence of negative polarity items (NPIs), but disappeared in the presence of NPIs. This raises further questions on how different speaker commitment scales interact and why.

## Introduction

Attitudinal expressions conveying speaker’s beliefs or preferences are pervasive in natural language and communication. However, the related expressions can pose challenges to formal theories of grammar due to pragmatics (e.g., multidimensionality, context-dependence, and subjectivity) and gradience. Their formal modeling presupposes an empirically adequate characterization, for which experimental methods are useful, and sometimes, indispensable. This paper reports on a case study of German conditional connectives (CCs), as those in (1)^1^. While conditionals are one of the most studied topics in cognitive science and linguistics, CCs have drawn attention to a much lesser extent than the other related lexical and grammatical devices. In the formal semantic literature, CCs as the English *if* are claimed to have no semantics in Kratzer’s (1991) restrictor analysis of conditionals. The existing vast linguistic literature on the interpretation of conditionals (to just name a few, e.g., Iatridou, 1991; von Fintel, 1999, 2007, 2011; Arregui, 2005; Grosz, 2012; Elder and Jaszczolt, 2016) shows effects of various factors (tense, mood, and polarity items) on the interpretation of conditionals, as well as the effect of CCs (e.g., Dostie, 1987; Léard, 1987 on CCs in French, Montoliìo, 2000; Schwenter, 2001 on CCs in Spanish, Ippolito and Su, 2014 on the Mandarin counterfactual CC *yaobushi* ‘if-not’, Hoeksema, 2012 on *unless* and among many others, also Declerck and Reed, 2001 on a comprehensive analysis of conditionals in English and Breindl et al., 2014 on connectives in German).


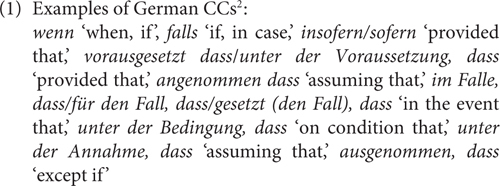


As is known from the literature, conditionals are non-veridical (Giannakidou, 1998, 1999), that is, *if-*clauses do not entail the truth of the antecedent proposition. In addition, the literature also shows that the non-veridicality property of conditionals can be influenced by various factors. The first, and probably most studied, is tense and mood choice, which reflects subjective (non-veridical) judgments. Conditionals in languages with tense and mood morphology come in two sorts: indicative and subjunctive. While the former is non-veridical, the latter is antiveridical, i.e., it presupposes (or implicates) the falsity of the antecedent proposition. That is, in (2a) the speaker does not know if John gets a promotion or not, but in (2b) the speaker presupposes that John did not get a promotion^3^.


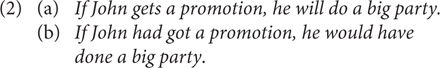


CCs, just as tense or mood choice, can reflect the speaker’s doxastic assumptions at semantic and pragmatic levels. In this paper, I will use distributional and experimental evidence to argue that apparently similar CCs differ in lexical pragmatics (see Visconti, 1996 on CCs in Italian; Liu, 2019; Liu and Wang, 2021 on CCs in Mandarin)^4^. More specifically, they can express different degrees of credence toward the modified proposition. The meaning difference between various CCs in this regard can be formally modeled using speaker commitment^5^ scales (Giannakidou and Mari, 2016) and as non-at-issue meanings (Simons et al., 2010) or, more precisely, an implicature resulting from the lexical choice between similar CCs. The paper focuses on the German CCs *wenn* vs. *falls*. It is organized as follows: Section “Non-at-Issue Meanings of *wenn/falls* in German” presents the distributional properties of *wenn/falls*, and provides an analysis relating *falls* to a weakened speaker commitment in contrast to *wenn*. Section “Experiments” reports on four experiments testing the analysis. Section “General Discussion and Conclusion” discusses the results and concludes the paper.

## Non-at-Issue Meanings of *wenn/falls* in German

In German, *wenn* is a more frequent word than *falls*^6^, but researchers do not have a consensus regarding the question whether *wenn* or *falls* is the prototypical CC. The handbook of Breindl et al. (2014) contains a comprehensive description of the German CCs in comparison to one another and also to other connectives. I will not go through the entire list, which also includes the discussion of *wenn/falls-*complement clauses, irrelevance conditionals (*selbst/auch wenn/^∗^falls* ‘even if’) and except-conditionals (*außer wenn/falls* ‘except if’). The authors also discuss the availability of causal and concessive readings for *wenn* but not for *falls*, which I will not deal with in this paper as the semantic or pragmatic status of the causal inference in indicative conditionals is debatable (see Volodina, 2006, 2011; Krzyżanowska et al., 2017; Krzyżanowska, 2019; Skovgaard-Olsen et al., 2016), as well as that of the concessive reading. In a nutshell, syntactically speaking, *wenn* and *falls*, by and large, have similar distributions in terms of syntactic positions where they can occur, but there is a preference for *wenn* over *falls* in adverbial clauses in a sentence-final position (Breindl et al., 2014). Semantically, the most prominent difference between them lies in that *wenn* has both a conditional and a temporal reading^7^ whereas *falls* only has a conditional reading, which makes the use of the latter more restricted. Furthermore, Volodina (2006) relates their meaning differences to factivity and specificity: the non-factive specific use of *wenn* gives rise to ambiguity between a temporal (similar to *sobald* ‘as soon as’) and a conditional reading (similar to *falls*), see (3a); a non-factive generic use is possible for *wenn* but *falls* only allows a specific use, see (3b).


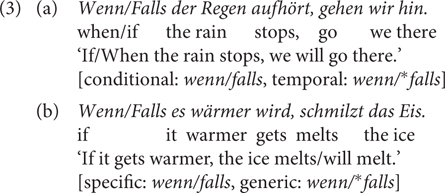


In the following of this section, I will present additional distributional properties of *wenn* and *falls* in different kinds of conditionals (von Fintel, 2007, 2011) and provide an analysis capturing their contrast.

### Distribution of *wenn* vs. *falls*

Both *wenn* and *falls* are fine in indicative conditionals (3) and biscuit conditionals (which assert the consequent proposition with no conditional dependence on the antecedent), see (4).


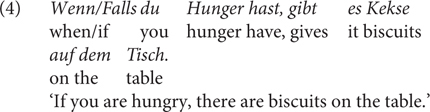


But they differ, among others, in the following aspects. The first contrast (Contrast 1) is that *wenn* can, but *falls* cannot, be used in premise conditionals, such as in (5), which presupposes that someone other than the speaker, in this case A, believes the truth of the antecedent proposition (Iatridou, 1991). The speaker accommodates the presupposition by using *wenn*, for which *falls* is odd^8^. The same contrast holds for factive conditionals as in (6a), with the speaker or contextual presupposition that the antecedent is true, or (6b) from Breindl et al. (2014, p. 756). However, for the latter case, it seems more appropriate to translate the *wenn-*sentence using *since*; this point has been made in Volodina (2006, pp. 367, 368) who claims that a factive use of *wenn* does not allow a purely conditional reading or a temporal reading, but can receive a causal interpretation.


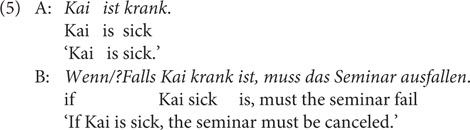



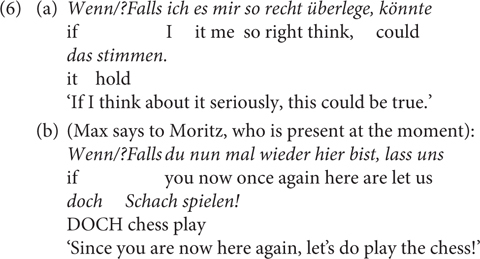


The second – controversial – contrast (Contrast 2) is that *falls* is degraded in counterfactual conditionals (indicated by subjunctive mood in German, henceforth “subj” in the examples) or less preferred than *wenn*, see (7). For example, according to the “grammis”^9^, counterfactual use of *falls* is usually excluded, with some exceptions, as in their example (8) below. However, it is to note that there might be regional differences in this regard: Some native speakers I consulted with do not judge (7) with *falls* to be degraded.


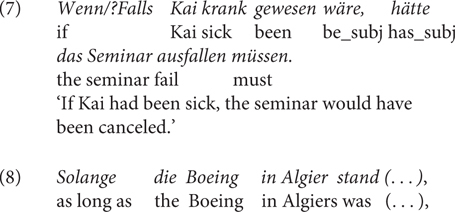



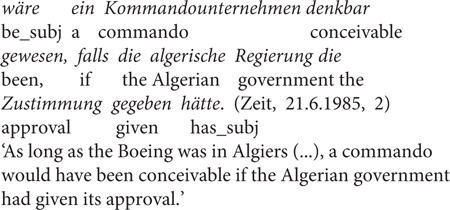


The third, uncontroversial contrast (Contrast 3) is that *falls* is out in counterfactual optatives, see (9). Following Grosz (2012), I assume that counterfactual optatives have no descriptive but presuppositional and expressive content, as illustrated below.


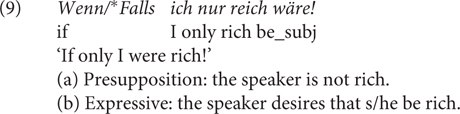


The fourth contrast (Contrast 4) is that unlike *wenn, falls* is degraded with the quantifying adverb *immer* ‘always’ (Zaefferer, 1991). For the minimal pair in (10a), Zaefferer (1991, p. 216) argues “that explicitly quantified c-constructures are plural forms, bare c-constructures with particles like *if* are transnumeral forms (unspecified with respect to number), and that bare c-constructures with *falls* are singular forms.” Some speakers pointed out to me that the only, or the more prominent, reading of (10a) is temporal; this is in line with the claim made in Breindl et al. (2014, pp. 765, 766) that unlike *wenn, falls* cannot quantify over time points but is used only to hypothesize based on the truth or falsity of the antecedent proposition. However, as shown in (10b), the prominent reading of the sentence is clearly conditional, that is, the adverb quantifies over cases rather than times. In Section “Non-at-Issue Meanings of *wenn/falls* in German,” I will argue instead that the contrast is due to the presupposition of *always*, which clashes with the meaning of *falls.*


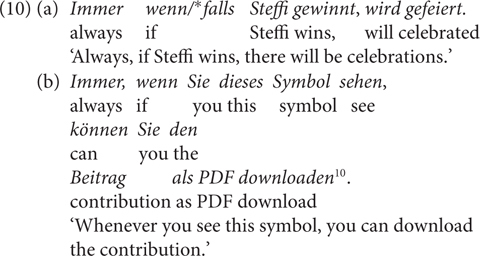


Last but not least, while both *wenn/falls* license NPIs, such as *auch nur irgendein* ‘even any’ in (11), Liu (2012) claims that *falls* is degraded with factive evaluative adverbs, which show PPI (positive polarity item) behavior (Contrast 5), see her example in (12). The speakers I checked with have different intuitions about *wenn* in (12): It is fine for some, and for others, it is equally odd as *falls*.


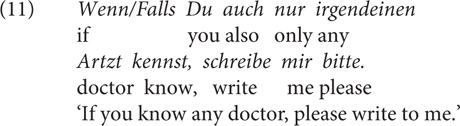



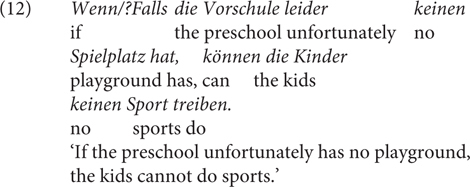


Below, I will provide an analysis to account for the *wenn/falls* contrast based on the above observations.

### Analysis

In the analysis, I will use two theoretical components: One is the speaker commitment scales used in Giannakidou and Mari (2016, 2021). Following their works, I assume “non-veridical equilibrium” (implying that p and ¬p as equal possibilities) to be the default for epistemic possibility, questions, and conditionals. That is, the speaker does not convey any preference for p or ¬p. But the equilibrium of conditionals (as for questions) can be manipulated to produce bias (i.e., reduced or higher speaker commitment) through various lexical or grammatical devices (for German, see Reis and Wöllstein, 2010; Liu, 2019; Sode and Sugawara, 2019; Liu et al., 2021). In the following, I will provide several examples as triggers of speaker bias and then argue that the *wenn/falls* contrast can be captured along the lines. The other component is the notion of non-at-issue meanings (e.g., Simons et al., 2010; Tonhauser, 2012). I will argue using diagnostic tests from the theoretical literature that the speaker bias conveyed by *falls* in comparison to *wenn* is a non-at-issue meaning. While non-at-issue meanings can be semantic or conventional such as conventional presuppositions or conventional implicatures (Potts, 2005, a.o), I will show further that the non-at-issue of *falls* is of conversational nature as well as that the implicature is different from scalar implicatures.

Giannakidou (1998, 2014), in her (non)veridicality framework, has related attitudes (i.e., speaker’s doxastic assumptions) to the notion of speaker commitment. In more recent works, Giannakidou and Mari (2016) argue that differences of attitudes can be modeled through speaker commitment scales (SCSs). For example, they apply the scale in (13) to capture the speaker’s doxastic attitude toward the modified proposition. The necessity modal verb *must* conveys a stronger speaker commitment than the possibility modal adverb *possibly*, but a weakened speaker commitment in comparison to the unmodalized variant, which expresses full commitment.


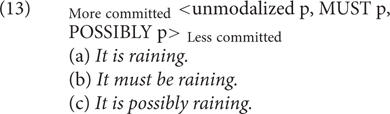


It has to be further explored whether the SCSs can encode not only doxastic attitudes but also deontic or bouletic ones, and whether the perspectival agent must be the speaker or can be a sentence subject or another discourse referent. Furthermore, Giannakidou and Mari (2016) remain non-committed as to the semantic or pragmatic nature of this meaning difference, namely, whether it is at-issue or non-at-issue, and in the latter case, whether it is a conventional implicature or a conversational implicature. For example, while the weaker speaker commitment meaning of the possibility modal verb seems to be its semantics, it is unclear how the weakened speaker commitment meaning of *must* relates to its necessity modal meaning. It seems that alternatives and their commitment strength of SCSs are determined by a variety of factors ranging from the at-issue as well as non-at-issue content. Thus, these scales might be different from Horn scales based on entailment relations. With these open questions kept in mind, I will show that SCSs are very useful for modeling grammar of speaker commitment in general, and provide experimental work testing these in Section “Experiments.”

First, for example, Zimmermann (2004) argues that the German discourse particle *wohl* (roughly ‘probably’) expresses a higher speaker credence in the truth of the proposition than the possibility adverb *vielleicht* ‘possibly.’ Thus, we can fit these alternations into a SCS in (14).


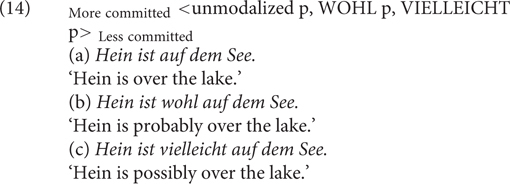


Second, Liu (2012) argues to distinguish between factive and non-factive evaluative adverbs in German, which show different distributions in entailment-canceling contexts (Simons et al., 2010). For example, *leider* and *unglücklicherweise* both mean roughly ‘unfortunately,’ but the latter can occur in questions, conditionals and modals, whereas the former is odd in these contexts. Liu (2012) thus labels *leider* as a factive adverb and *unglücklicherweise* a non-factive one. This idea can be equally translated into a SCS as in (15).


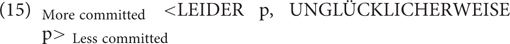


Third, Liu (2019) shows experimental evidence that in German and English conditionals, NPIs (*jemals/überhaupt* ‘ever/at all’) led to lower ratings of speaker commitment to the antecedent proposition in comparison to sentences without NPIs. This finding can be put into the SCS in (16). Whether this scale holds for all NPIs or not is a question beyond the scope of this paper.


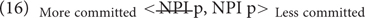


Fourth, SCSs can also be used to model the difference between the clausal connectives. For example, in contrast to non-veridical CCs, causal connectives are veridical or factive operators, that is, they convey the speaker’s full commitment to the truth of the antecedent (Giannakidou, 1998, et seq). This idea can also be put into a SCS, as shown in (17).


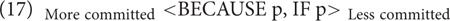


In the rest of this section, I will argue that SCSs are also useful for modeling the internal differences among the CCs.

That CCs can differ in degrees of speaker commitment is not new. For example, Visconti (1996, p. 555) claims that CCs can contribute secondary (in recent terms, ‘non-at-issue’) meanings concerning a ‘propositional attitude’ toward the modified propositions, such as the speaker’s epistemic/doxastic/deontic/emotional evaluation toward the antecedent or the consequent. In Italian, Visconti claims that the CCs *nel caso che* ‘in the case that,’ *nell’eventualità che* ‘in the eventuality that’ and *casomai* ‘if-ever’ [made up of a simple CC *caso* ‘in case, if’ and a NPI *mai* ‘ever’] differ in terms of the speaker’s attitude toward the antecedent ‘p’ that is expressed at the level of conventional implicatures: While *nel caso che* is doxastically neutral, *nell’eventualità che* expresses a negative bias ‘unlikely(p)’ and *casomai* conveys an even stronger bias, namely, ‘improbable(p).’ Due to the different degrees of the bias, it is odd to use *nell’eventualità che* (or *casomai*) for modifying the antecedent that is simultaneously labeled as highly likely by the non-restrictive relative clause, whereas it is not a problem for *nel caso che*, as shown in her example (18) (Visconti, 1996, p. 559). The idea can be translated into a SCS in (19).


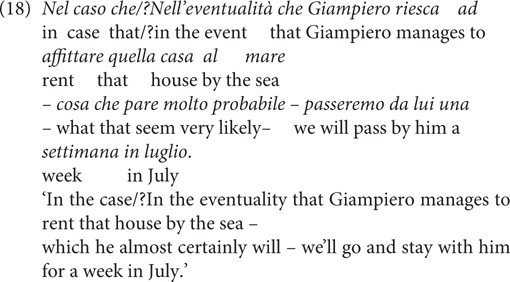



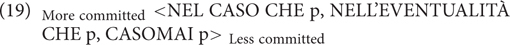


Visconti’s proposal of treating the speaker assumption conveyed by these CCs as conventional implicature is a very insightful idea and is obviously useful for analyzing complex (i.e., multi-word) but compositional CCs (i.e., with transparent semantics which can be derived compositionally with the subparts of the CC) such as those in (20). The adjectives provide information about the speaker’s doxastic (20a) or bouletic assumptions (20b) about the antecedent proposition. These meanings are logically and compositionally independent of the conditional core in these sentences, and thus are indeed conventional implicatures in the sense of Potts (2005). On the other hand, conventional implicatures are neither cancellable nor reinforceable, compared to conversational implicatures, which are cancellable and reinforceable. This raises the empirical question whether all the CCs express speaker bias at the dimension of conventional implicatures, or whether they can encode weaker, i.e., non-conventional, meaning.


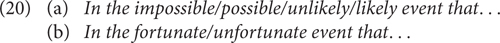


Liu and Wang (2021) provide distributional and experimental evidence that the Mandarin Chinese CC *wanyi* (lit, ‘one of ten thousand,’ originally a numerical expression, used as a CC in modern Mandarin) conveys a weakened speaker commitment than *ruguo* ‘if’, as in (21)/(22). They treat this meaning difference at the dimension of non-at-issue-meanings (Simons et al., 2010).


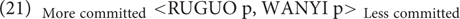



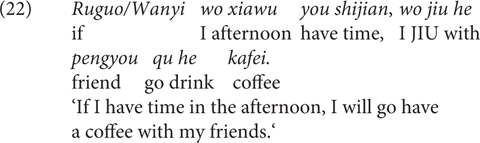


The same scale can apply to the German CCs, such as in (23a): *wenn/falls* express speaker commitment of intermediate degree between *im wahrscheinlichen Fall, dass* ‘in the probable event that’ and *im unwahrscheinlichen Fall, dass* ‘in the improbable event that.’ Further, I argue that compared to *wenn, falls* expresses a weakened speaker commitment toward the antecedent proposition (*p*), see (23b). That is, *falls* indicates that the speaker takes *p* as not likely. This meaning acts at a separate layer of doxastic states, i.e., it does not target the question under discussion, and thus it is non-at-issue (Simons et al., 2010).


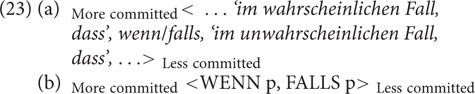


Following this, *falls* has an attitudinal meaning at a separate layer of doxastic states, i.e., λp.¬likely(*p,x*) with x as a free variable (for the attitudinal holder) whose value is to be determined by context (e.g., *x* is the speaker, or the sentential subject). This is what I call ‘weak unlikelihood implicature’ (WUI). A sentence such as (24) expresses an at-issue content as proposed by Kratzer (1986, 1991) and paraphrased in (24a), and additionally, a non-at-issue content as in (24b).


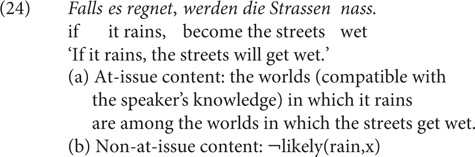


I will first address the non-at-issue and the implicature part of the proposal and then explain why it should be “weak.”

Tonhauser (2012) puts forward three criteria along which at-issue content differs from non-at-issue content: First, at-issue content can be directly assented or dissented with, but non-at-issue content cannot. Second, at-issue content addresses the question under discussion, but non-at-issue content does not. Third, at-issue content determines the relevant set of alternatives whereas non-at-issue content does not. I will apply one test Tonhauser proposes based on the first criterion in (25): As is shown, the conditional (i.e., at-issue) meaning in A’s utterance can be assented or dissented with positive continuation (B1 and B2) but the speaker assumption about the antecedent proposition cannot (B3 and B4). This contrast speaks in favor of the non-at-issue status or pragmatic nature of the bias encoded in *falls.*


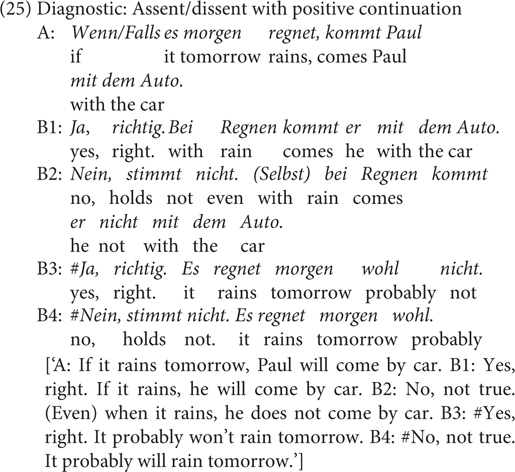


Furthermore, non-at-issue contents can project out of entailment canceling contexts (Simons et al., 2010; Liu, 2012). For example, in (26), the intuition is that the bias conveyed by *falls* survives embedding in the question operator, an entailment canceling context.


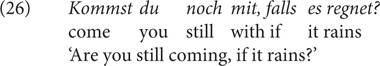


In addition, consider the minimal pair in (27) from Liu (2019): The relative clause in the sentence indicates the speaker’s commitment to the antecedent proposition, which does not go along with the CC *im unwahrscheinlichen Fall, dass* but is ok with *falls.* This indicates that in the former case, the unlikelihood meaning component is semantic/conventional and thus uncancellable, whereas it is pragmatic/conversational and thus cancellable in the latter case.


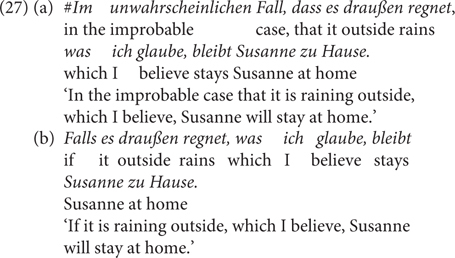


These data taken together indicate that the weak bias created by *falls* is a secondary, i.e., non-at-issue, content that is lexically triggered but contextually cancellable, and more precisely, an implicature (Grice, 1975; Potts, 2005; see Zakkou, 2018 for discussion of the reliability of the cancellability test). Here, a natural question arises whether it is a scalar implicature. However, if we compare the CC scale in (23b) with a typical Horn scale such as <all, some>, there are at least the following three aspects where they differ: First, the scalar implicature, e.g., *not all students came* is computed based on the semantics of the sentence *some students came*, whereas it is not the case for *wenn/falls p, q* as the negative bias does not target the conditional dependence between the antecedent and the consequent. Second, related to the first aspect, the bias by *falls* conveys speaker’s assumptions that the hearer can ignore, as it does not target the QUD whereas scalar implicatures can target QUDs, e.g., *How many students came?* in the above example. The third and most straightforward argument against a scalar implicature analysis for the *wenn/falls* contrast is that the Horn scale is based on a proper entailment relation, e.g., *all students came* entails *some students came*, whereas this does not hold for *wenn p, q* and *falls p, q*: semantically, they both convey the same conditional relation between *p* and *q* in that all *p*-cases are *q*-cases. Thus, I take the implicature by *falls* to be different from scalar implicatures.

The naturally occurring examples in (28) show the speaker’s awareness of the meaning difference between *wenn* and *falls*. Whereas *wenn* in (28a) can have either a conditional or a temporal reading, (29) is unambiguously meant as a conditional^11^.


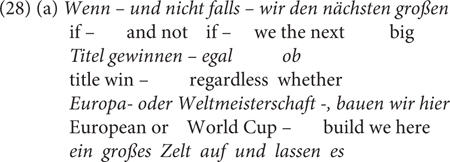



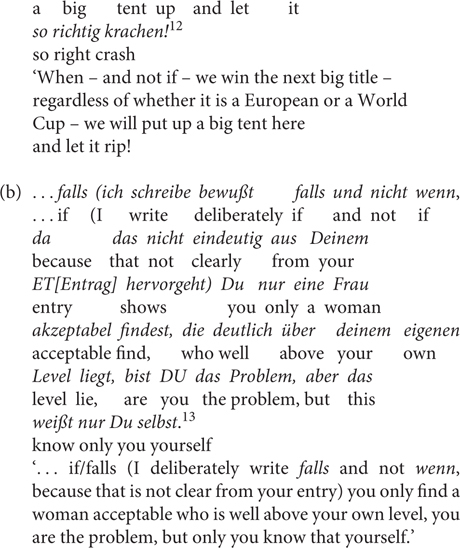


I argue that *falls* encodes a weak unlikelihood meaning (i.e., that the speaker does not take the antecedent proposition as likely) instead of a strong unlikelihood meaning (i.e., that the speaker takes a proposition as unlikely). The latter meaning is expressed by, for example, additive particles such as the English *even*. As shown in (29), a strong unlikelihood meaning will be too strong for *falls.*


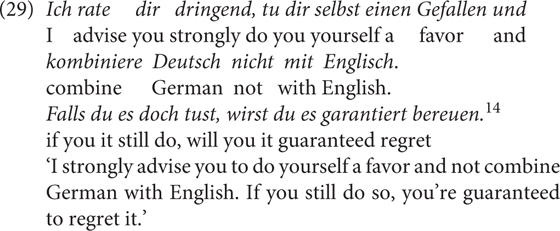


By this analysis, the degradation of *falls* in premise/factive conditionals (Contrast 1) results from the clash between the speaker presupposition and the WUI of *falls. Falls* is degraded in counterfactual optatives and arguably in counterfactual conditionals (Contrast 3 and 2) due to the counterfactual presupposition or implicature (i.e., speaker’s anti-commitment to the antecedent proposition). This contradicts the meaning of *falls*, which presupposes the absence of bias to start with, i.e., the non-veridicality of the antecedent proposition (dubbed as the “Non-veridicality Equilibrium,” the default of epistemic possibility, in Giannakidou and Mari, 2021), and implicates the WUI. The reason why *falls* is acceptable in counterfactual conditionals for some speakers might be because the negative bias of *falls* is a conversational implicature (which is lexically triggered but needs contextual support) and thus cancellable or optional (Grice, 1989; Zakkou, 2018), or in general, there might be individual differences in the (quality of the) lexical representations of *wenn/falls.* The latter goes far beyond the scope of this paper and thus will not be addressed here. Another possibility is that subjunctive conditionals do not always presuppose or implicate the falsity of the antecedent proposition. But this is not a plausible explanation, as even though the counterfactuality inference does not always hold, it cannot be canceled without good reason (see Footnote 3 and the cited references therein).

For Contrast 4, I propose an alternative explanation to Zaefferer’s (1991) account, namely, the wide-scope *immer* is presuppositional. It presupposes that the event in the antecedent takes place more than once, which clashes with the WUI by *falls.* For illustration, *immer* in (10) presupposes that Steffi wins more than once, but *falls* would convey that it is not likely that Steffi wins, thus their combination is odd. The degradation of *falls* in co-occurrence with factive adverbs (arguably compared to *wenn*, see Contrast 5) can also be attributed to the factivity presupposition of the adverb, in clash with its WUI. All in all, this shows that the proposed difference in terms of speaker commitment for *wenn/*falls is able to account for the listed distributional differences: *falls*, as the more restricted CC in comparison to *wenn*, has the proposed lexical pragmatics, which is cancellable and reinforceable through grammatical devices, as we will see in Section “Experiments.” I leave it open for now whether some of the differences can be captured differently, but will discuss several alternative accounts, which I argue are in line with the proposed one.

The present analysis for *wenn/falls* echoes the observation occasionally made in the previous literature, for example, by Breindl et al. (2014, pp. 114, 115): “The difference between *wenn* and *falls* has to do with the probabilities of the occurrence of the antecedent” (translated from German). They use the example in (30) to argue that the speaker, being aware of their differences, uses one CC or the other to indicate implicitly their assessment of the probability of the antecedent proposition (i.e., speaker commitment).


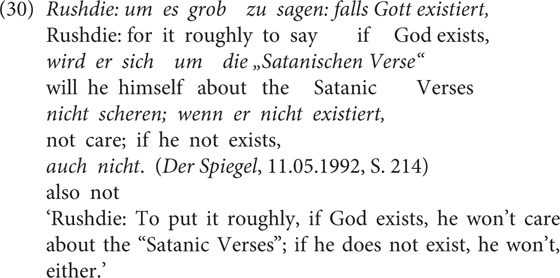


So far, we have seen distributional properties of the *wenn/falls* contrast and I proposed that the two CCs differ in degrees of conveyed speaker commitment. In the following, I will report on experimental work that tested the proposed contrast.

## Experiments

I conducted three experiments to test the proposed analysis above, as well as a fourth experiment on the interaction between two different SCSs. In addition, I will also report on the related German experiment from Liu (2019).

### Experiment 1

The original assumption for Experiment 1 was that if *falls* carries a negative bias in comparison to *wenn*, as illustrated in (31), then it might tend to occur more naturally with events (or, propositions) that are less likely, i.e., more naturally with p3 than with p1 in (32). This assumption turned out to be problematic. I report on this experiment here nevertheless, as it is important to showcase potential pitfalls and necessary “precautions” to take for testing lexical pragmatics in general.


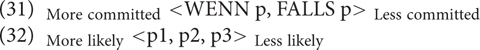


#### Materials and Methods

Experiment 1 was a rating study based on a 2 × 3 within-subjects design, with one factor being CC (*wenn/falls*) and the other factor being the likelihood of the event in the conditional antecedent *p* (in three levels, i.e., very likely/likely/unlikely), see Table 1. The starting prediction was that due to the weakened speaker commitment by *falls* in contrast to *wenn*, it might be degraded in combination with very likely and likely events in comparison to the other conditions.

**TABLE 1:** Factors, conditions and predictions of Experiment 1.

Events	*wenn*	*falls*
Very likely	1: (a)	2: ## (b)
Likely	3: (c)	4: ^#^(d)
Unlikely	5: (e)	6: (f)

Twenty-four items were used, with one example in (33), as well as 84 additional filler items. The critical stimuli are provided in the Supplementary Materials (Test sentences of Experiment 1)^15^.


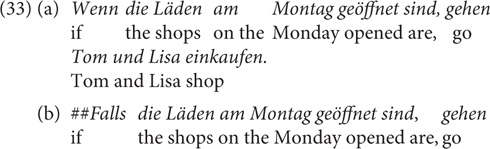



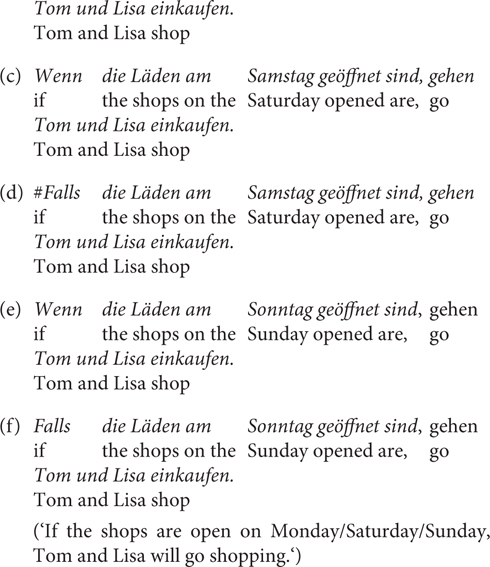


As the German CC *wenn* has both a temporal (i.e., *when*) and a conditional (i.e., *if*) reading in present indicative, a temporal adverb was used in the antecedent so that the conditional reading of *wenn* becomes more plausible than the temporal reading. The focus was on whether the naturalness of *falls* decreases by the likelihood increase of *p*. The events were chosen and ordered based on common knowledge in the German context. They were tested informally with 10 subjects (undergraduate students of Osnabrück University). The subjects saw a list of three-event pairs (e.g., the shops are open on Monday/Saturday/Sunday) in different orders and were asked to order the events in each pair by their likelihood. The results show that the scales were all valid^16^.

The experiment was conducted online using the SoSci Survey^17^. 36 undergraduate students (25 female, 11 male; 35 between 18 and 29 years old, 1 under 18)^18^ of Osnabrück University took part in the study online for course credits. The participants each saw 108 sentences in total, which were presented one by one in the middle of the computer screen, and they rated the naturalness of each sentence (0: unnatural, 1: natural). Our predictions were that (33b/d) would receive lower ratings than (33a/c/e/f), as the negative bias by *falls* might clash with the likely events of the shops being open on Monday or Saturday in the German context.

#### Results

All analyses were performed using mixed effects linear regression models. The models were constructed using the *lme4* package (Baayen et al., 2008; Bates et al., 2012) in R (R Core Team, 2018). The reported model is the maximal model that converged (Barr et al., 2013). The model included CC and Event-likelihood (with interaction term) as fixed effects. Furthermore, it included random intercepts for subjects, items and stimuli order, as well as random by-subject and by-item slopes for the effects of CC and Event-likelihood.

**FIGURE 1:**
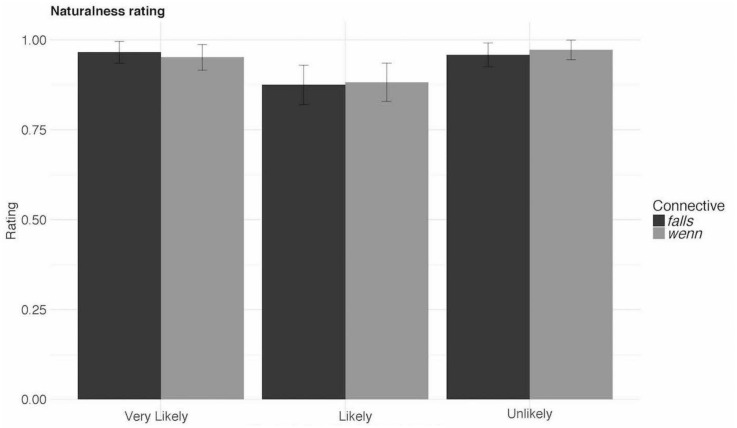
Results of Experiment 1.

The results (see Table 2 and Figure 1) showed neither the interaction nor the main effect for CC. That is, the comparison between *wenn* and *falls* was not significant (*t* = 0.15, *p* = 0.88). For the Event-likelihood, there is a numerical difference in the naturalness rating for likely events when compared to either unlikely or very likely events. However, these contrasts, too, fail to reach significance [Tukey’s test for multiple comparisons of means: *t* = 1.52, *p* = 0.26 (very likely vs. likely); *t* = 1.63, *p* = 0.215 (unlikely vs. likely)].

**TABLE 2:** Descriptive statistics of Experiment 1.

Condition	CC	Event	Rating	*SE*
1	*wenn*	Very likely	0.95	0.02
2	*falls*	Very likely	0.97	0.02
3	*wenn*	Likely	0.88	0.03
4	*falls*	Likely	0.88	0.03
5	*wenn*	Unlikely	0.97	0.01
6	*falls*	Unlikely	0.96	0.02

#### Discussion

Experiment 1 did not confirm the prediction and thus does not provide evidence for the proposed account of the *wenn/falls* contrast. This can mean that the two connectives are interchangeable in the conditional use. Alternatively, the methods used here might be problematic. First, some of the fillers involve unlicensed polarity items, e.g., *jemals* ‘ever’ in positive sentences, which are ungrammatical (see Ladusaw, 1980; Giannakidou, 2011; Liu et al., 2019). This might have contributed to the ceiling effect for the *wenn/falls* sentences. Second, the attitude by *fall*s is a speaker-oriented (i.e., subjective) meaning at a separate dimension (i.e., non-at-issue), i.e., it can be ignored from the hearer’s (i.e., subjects’) perspective. In other words, the contrast is context-dependent, i.e., present in some contexts and absent in others (as Experiments 2–4 will show). Thirdly, the naturalness rating studies with the binary scale was maybe not sensitive enough to measure such subtle lexical pragmatics (e.g., due to shallow processing).

### Experiment 2

Experiment 2 used the forced lexical choice paradigm. It was based on the assumption that if the *wenn/falls* contrast was real, then, given both as possible lexical items to use, speakers would opt for one or the other in a conditional expression depending on the context, i.e., their degree of commitment or credence in the antecedent. More specifically, in a context where the protagonist is positively biased toward the antecedent proposition, they would use *wenn* more often than *falls;* in a context where the protagonist is negatively biased toward the antecedent proposition, a reverse pattern is to be expected, see Table 3.

**TABLE 3:** Factor, conditions, and predictions of Experiment 2.

Context	*wenn* vs. *falls*
Belief-context	*wenn* preferred to *falls*
Disbelief-context	*falls* preferred to *wenn*

#### Materials and Methods

Experiment 2 was based on a one factorial within-subjects design with two levels for the factor CONTEXT, encoded in the sentence preceding the conditional sentence. Bearing in mind that the negative bias of *falls* is subjective meaning, I used a third-person protagonist to keep the doxastic anchoring constant, i.e., to prevent subjects from taking egocentric perspectives. The protagonist either believes *p* or not.

The subjects were asked to choose among *wenn, falls* and a mismatching control item such as *oder* ‘or.’


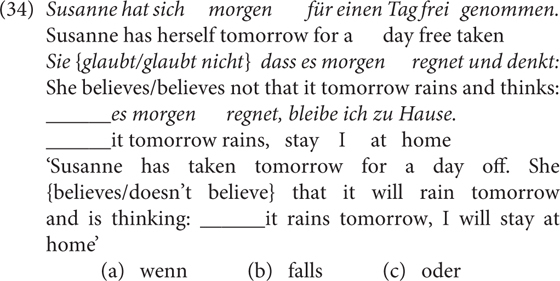


Twenty-four items such as (34) were used, as well as 48 fillers. The critical stimuli are provided in the Supplementary Materials (Test sentences of Experiment 2). The experiment was programmed in Python and conducted in the behavorial lab of the Institute of Cognitive Science of Osnabrück University. 52 undergraduates (29 female, 23 male; mean age = 21.2, *SD* = 1.7) of Osnabrück University participated in the study for course credits.

#### Results

Wrong answers, i.e., answers with the mismatching lexical items [e.g., *oder* ‘or’ in (34)], were excluded from the data analysis. The response (see Table 4) in this experiment is binary, not numeric (i.e., a binary choice between *falls* and *wenn*), therefore all analyses were performed with mixed logistic regression models. The model included the answer choice (*falls/wenn*) as dependent variable and the CONTEXT (belief/disbelief) as predictor variable. The random effects structure included random intercepts for subjects, items and stimuli order, as well as random by-subject slopes for the fixed effect. The model reported is the maximal model that converged. The model has a good fit (precision = 0.79, recall = 0.78) and performs significantly better than a baseline of guessing a response (*p* < 0.0001). It yields a significant effect of CONTEXT (*t* = 6.19, *p* < 0.0001), that is, subjects are significantly more likely to choose *falls* under the disbelief-condition and *wenn* in the belief-condition.

**TABLE 4:** Results of Experiment 2.

Context	*wenn*	*falls*
Belief-context	442	185
Disbelief-context	177	436

#### Discussion

Experiment 2 shows that the doxastic agent chose *falls* over *wenn* if they have a low degree of credence toward the modified antecedent proposition. As the materials use both CCs in the sentence-initial position, I take the results to reflect meaning differences instead of syntactic preferences (in a sentence-final position, as Breindl et al., 2014 point out). That is, they provide positive evidence for the *wenn/falls* contrast in terms of degree of speaker commitment.

### Experiment 3

Experiment 1 did not confirm the *wenn/falls* contrast. As mentioned above, this can be due to the context-dependence of the contrast due to, among others, the subjective (speaker-oriented) nature of the bias encoded in *falls*. For this reason, Experiment 3 included an additional relative clause commenting on the conditional antecedent. The rationale was that in this way, the attitudes encoded in the CC and the relative clause had the same anchoring toward the speaker. While this was also controlled in Experiment 2, Experiment 3 was conducted to find out whether the bias is cancellable (by a RC conveying speaker’s disbelief in the antecedent proposition) or reinforceable (by a RC conveying speaker’s belief in the antecedent proposition). The results can shed further light on the nature of the bias. If the meaning difference is cancellable and reinforceable, we can conclude that it is a pragmatic difference.

#### Materials and Methods

Experiment 3 was a rating study based on a 2 × 2 factorial within-subjects design, with the factor CC (*wenn/falls*) and RC (relative clause) expressing a likelihood/unlikelihood propositional attitude toward *p*. The method and procedure were similar as in Experiment 1. 24 items were used as well as 72 fillers. Half of the items used RCs as in (35) and the other half used RCs as in (36). The critical stimuli are provided in the Supplementary Materials (Test sentences of Experiment 3). We did not control the likelihood of the antecedent and used the unlikely conditions due to lack of contrast for this manipulation in Experiment 1. 40 undergraduates (25 female, 15 male; 39 between 18 and 29 years old, 1 between 30 and 39) of Osnabrück University participated in the experiment for course credits.


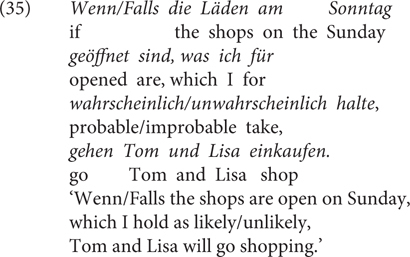



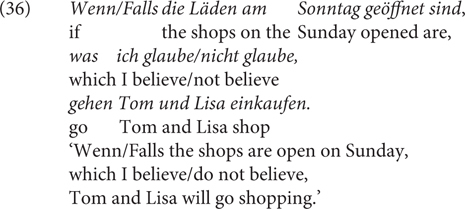


#### Results

All analyses of the data (see Table 5 for descriptive statistics) were performed using mixed effects linear regression models. The models were constructed using the *lme4* package in R (Baayen et al., 2008; Bates et al., 2012; R Core Team, 2018). All contrasts of interest, i.e., CC and RC, were sum coded and included as fixed effects in the models. The reported models are the maximal models that converged.

**TABLE 5:** Descriptive statistics of Experiment 3.

Condition	CC	RC	Rating	*SE*
1	*wenn*	Belief	0.78	0.05
2	*wenn*	Disbelief	0.73	0.06
3	*falls*	Belief	0.73	0.06
4	*falls*	Disbelief	0.79	0.05

**(A)** Descriptive statistics of Experiment 3 with the RC *was ich glaube/nicht glaube* ‘which I believe/do not believe.’				

1	*wenn*	Belief	0.66	0.04
2	*wenn*	Disbelief	0.66	0.04
3	*falls*	Belief	0.77	0.04
4	*falls*	Disbelief	0.82	0.04

**(B)** Descriptive statistics of Experiment 3 with the RC *was ich für wahrscheinlich/unwahrscheinlich halte* ‘which I deem likely/unlikely.’				

1	*wenn*	Belief	0.91	0.03
2	*wenn*	Disbelief	0.81	0.04
3	*falls*	Belief	0.70	0.04
4	*falls*	Disbelief	0.76	0.04

The first model included CC and RC (with interaction term) as fixed effects. Furthermore, it included random by-subject and by-item intercepts, as well as random by-subject and by-item slopes for the effects of CC and RC (and their interaction). Neither of the two main effects was significant. There was a significant interaction between CC and RC (*t* = 2.15, *p* = 0.03): in the belief-condition, *wenn* was rated more natural than *falls*. The reverse pattern was found in the disbelief-condition. Pairwise comparisons between all four conditions, however, showed no significant differences between either of them, indicating that the interaction effect is highly nuanced.

Furthermore, the RCs in the experiment included 12 items with *was ich für wahrscheinlich/unwahrscheinlich halte* (‘which I deem likely/unlikely’), and another 12 with *was ich glaube/nicht glaube* (‘which I believe/do not believe’). Thus, I did an additional test to see whether and to what extent there were differences between the two types of predicates. First, a model was constructed which included the type of the predicates (henceforth, PREDICATE) as a third fixed effect and included a three-way interaction term between PREDICATE, CC, and RC. The random effects structure was identical to that of the first model. The new model indicates a significant effect for PREDICATE (*t* = 2.60, *p* = 0.009), but no other main effects or higher-order interactions between PREDICATE and CC or RC. However, the reader should keep in mind that the factor PREDICATE was not a systematically controlled condition within items. Thus, we cannot exclude the possibility that any effect was due to confounding influences that rendered some items more natural than others. Nevertheless, it may serve as the first indication of an effect, which can be further explored in future studies.

**FIGURE 2:**
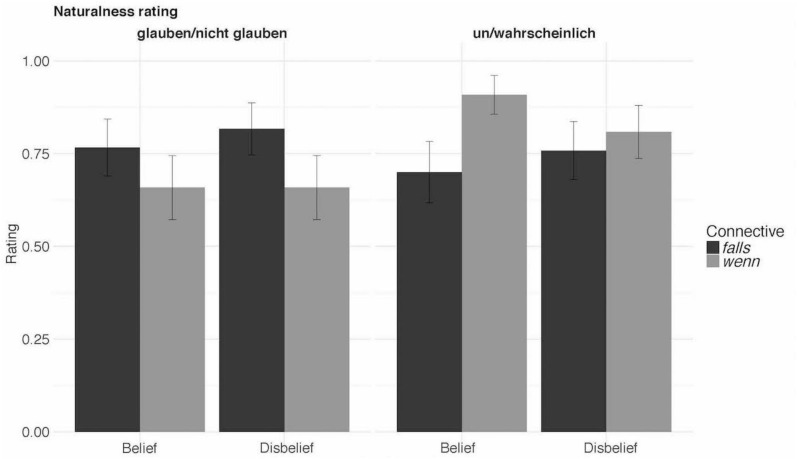
Results of Experiment 3.

To further explore the effect of the RC, separate models were created for the two sets of items, see Figure 2 and Tables 5A,B. Both models thus used half of the data set and included CC and RC (with interaction term) as fixed effects. Again, the random effects structure included random by-subject and by-item intercepts, as well as random by-subject slopes for the effects of CC and RC.

Firstly, for the items that used an RC with the verb *glauben/nicht glauben* ‘believe/not believe,’ no significant effects were found (see Table 5A). Neither the main effects nor the interaction turned out to be significant, that is, there was no systematic effect of either factor. For the items that used the RC containing *wahrscheinlich/unwahrscheinlich* ‘likely/unlikely,’ there was a significant interaction between CC and RC (*t* = 2.05, *p* = 0.04), see Table 5B. However, neither of the main effects turned out to be significant and paired-tests also showed no significant contrast between any of the comparisons, which indicates a high degree of variation in the data.

#### Discussion

The naturalness rating results of Experiment 3 are compatible with the lexical choice results of Experiment 2: *Wenn* is preferred over *falls* in the belief-context and vice versa in the disbelief-context. On the other hand, the results in Experiment 3 are also not straightforward to interpret.

As the analysis including PREDICATE shows, the overall interaction effect was mainly driven by the interaction among the items using the RC *was ich für wahrscheinlich/unwahrscheinlich halte* (‘which I deem likely/unlikely’). The *wenn*-sentences were rated more natural with these than with *was ich (nicht) glaube* (‘which I believe/do not believe’). A possible explanation for the effect of PREDICATE lies in their difference in terms of speaker commitment. As (37) shows, *was ich glaube/nicht glaube* (‘which I believe/do not believe’) conveys the speaker’s full commitment or anti-commitment, whereas *was ich für wahrscheinlich/unwahrscheinlich halte* (‘which I deem likely/unlikely’) conveys the speaker’s weakened commitment or weakened anti-commitment.


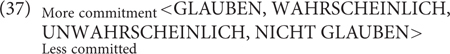


A full account of these differences presupposes a good understanding of the predicates *believe* and *probable* used in the RC, which goes beyond the scope of this paper. For example, it was pointed out to me (Juliane Schwab, p.c.) that the addition of certain adverbs improves the *believe*-sentences, as in (38). The effect of adding *durchaus* ‘quite’ weakens the speaker commitment, bearing a similar effect as *wahrscheinlich* ‘likely’. The addition of *eigentlich* ‘actually’ signals that there is a contextual expectation (e.g., of the shops being open) set by the antecedent which the speaker rejects with the use of the RC (see Bergena and Boskerb, 2018).


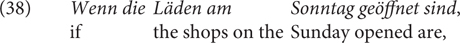



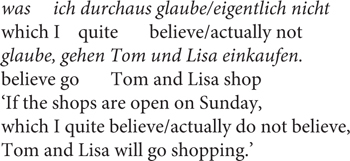


As to *falls*, it has been noted that the negative bias generated by it is not always cancellable, as can be seen in (39) from Liu (2019), attributed to an reviewer.


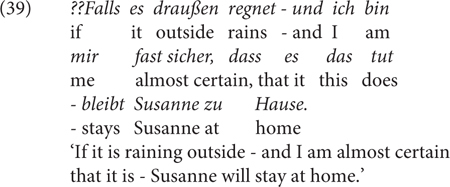


However, the native speakers I checked with have no problems with this sentence. Moreover, the data in Table 5A show no difference between the CC or the polarity of the RC. This means that the bias of *falls* is reinforceable (by the negative RC) and cancellable (by the positive RC). With the questions about the predicates (*believe, certain, likely*) left for the future, overall, Experiment 3 shows that the speaker bias encoded in *falls* is a conversational, non-at-issue meaning as is proposed in Section “Non-at-Issue Meanings of *wenn/falls* in German.” This is also in line with the results of Experiment 1.

### Experiment 4

Experiments 1–3 tested the *wenn/falls* contrast directly with different measures. The results were mixed with no evidence in Experiment 1, with evidence in Experiment 2 and with inconclusive results (weak evidence in the predicted direction) in Experiment 3. In this section, I report two additional experiments addressing the question of how the SCS of CCs interacts with other SCSs. Experiment 4a on the interaction of the CC scale and the NPI scale (16) refers to the German study reported in Liu (2019), which I summarize here. In comparison, Experiment 4b tested the interaction of the CC scale and the EDAV scale, as shown in (15). The NPI and the EADV scales differ from each other in that the former conveys weakened speaker commitment and the latter high speaker commitment, in line with their distributional requirements (NPIs for negative contexts and EADV for positive contexts). The purpose of these two studies is to reveal the CC contrast by checking their interaction with different SCSs.

#### Experiment 4a: Summary of Liu (2019)

In Liu (2019), the author reports on a “speaker commitment” rating experiment in German addressing the difference between *wenn/falls*, the effect of NPIs *jemals/überhaupt* ‘ever/at all,’ and their interaction. Subjects were given scenarios, e.g., (40), consisting of 4 sentences (S1–S4) presented one by one: S1 sets the context; S2 contains a conditional sentence in one of the four combinations, with half of them containing *jemals* and the other half *überhaupt* (e.g., *wenn-überhaupt, wenn*+*überhaupt, falls-überhaupt, falls*+*überhaupt*); S3 asks the subjects to rate the degree of the protagonist’s commitment to the antecedent on a 5-point Likert scale (1 = certainly not, 5 = certainly yes). S4 is a comprehension question. The results show a significant effect of CC (in that the *falls* conditions received lower ratings than the *wenn* conditionals), a significant effect of NPI (with high ratings in the conditions without NPIs than with NPIs) and a significant interaction. Both scales in (41) and (42) are confirmed, with a significant CC contrast in the absence of NPIs, which disappears in the presence of NPIs.


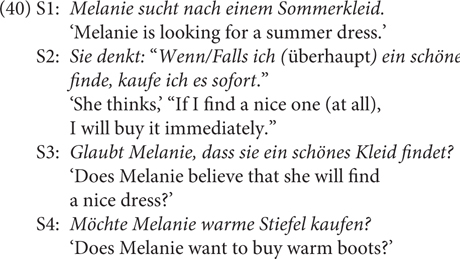



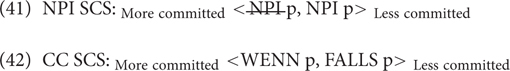


If Experiment 2 provides indirect evidence for the CC scale, the finding of this study complements it with direct evidence in favor of the *wenn/falls* contrast. But while Liu (2019) focuses on CCs and NPIs from a cross-linguistic perspective by comparing German vs. English, the current paper provides a more detailed descriptions and a set of experiments on *wenn/falls* with both theoretical and methodological implications.

#### Experiment 4b

Experiment 4b also tested the interaction between the CC SCS with another SCS, namely, by evaluative adverbs (EADV). Liu (2012) presents distributional facts of apparently similar EADVs in German and argues that they differ in terms of factivity. Factive EADVs occur only in veridical contexts, whereas non-factive EADVs are more tolerant, e.g., they can also occur in non-veridical contexts. Without going into detail, their distinction can be illustrated with (43). Both EADVs mean *unfortunately*, but in, for example, questions and conditionals (as non-veridical or entailment-canceling contexts), *leider* is degraded in comparison to *unglücklicherweise*, which Liu attributes to their difference in degrees of factivity, i.e., speaker commitment.


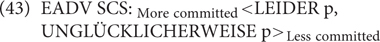


Experiment 4b, addressing the CC SCS and the EADV SCS, was based on the assumption that the degree of speaker commitment by one expression should be coherent with that of its co-occurring expression. Thus, both CCs should favor non-factive EADVs more than factive ones and factive EADVs should favor *wenn* over *falls* with no difference between the CCs in the case of non-factive EADVs.

#### Materials and Methods

Experiment 4b used a 3 × 2 factorial within-subjects design, with the factor CONNECTIVE (factive vs. non-factive, i.e., *weil* ‘because’ vs. *wenn/falls*) and EADV with the levels factive (e.g., *leider*) and non-factive (e.g., *unglücklicherweise*). 36 items such as (44) as well as 72 fillers were used. The critical stimuli are provided in the Supplementary Materials (Test sentences of Experiment 4b). The procedure was similar as in Experiment 1, except that the subjects gave naturalness ratings on a 5-point Likert scale (1 = unnatural, 5 = natural). 42 undergraduates (28 females, 14 males; 40 between 18 and 29 years old with 1 under 18 and 1 between 30 and 39) of Osnabrück University participated in the experiment for course credits.


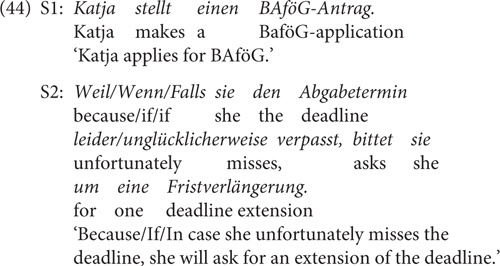


#### Results

All analyses were performed using mixed effects linear regression models. The model was constructed using the *lme4* package in R (Baayen et al., 2008; Bates et al., 2012; R Core Team, 2018). The reported model is the maximal model that converged. The model included CONNECTIVE and EADV (with interaction term) as fixed effects. Furthermore, it included random intercepts for subjects and items, as well as random by-subject and by-item slopes for the effects of CC and EADV.

**FIGURE 3:**
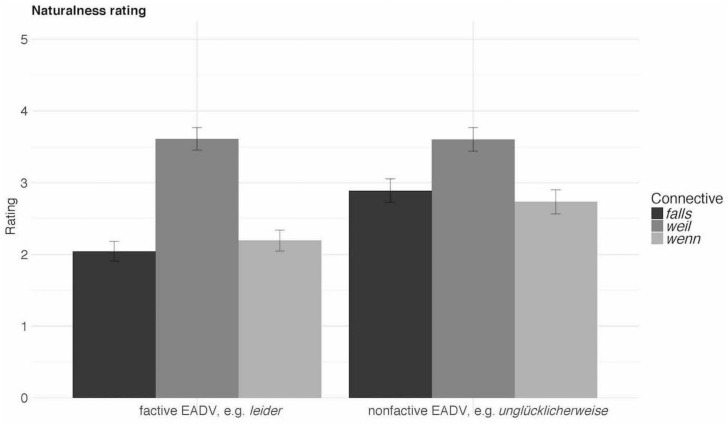
Results of Experiment 4b.

The results show a highly significant CONNECTIVE × EADV interaction (LRT = 56.92, *p* < 0.0001), see Table 6 and Figure 3. First, *weil*-sentences received significantly higher naturalness ratings overall than either *wenn-* or *falls*-sentences (*weil* vs. *wenn*: *t* = 8.19, *p* < 0.0001; *weil* vs. *falls*: *t* = 9.59, *p* < 0.0001), even though an reviewer pointed out rightly that the *weil*-sentences would have been more natural in present perfect (i.e., *verpasst hat* ‘has missed’). Second, for the causal connective *weil* ‘because’ both factive and non-factive EADVs were rated as equally natural. Third, non-factive EADVs were preferred over factive ones in the case of both CCs (*wenn: t* = 4.66, *p* = 0.0001, *falls: t* = 7.30, *p* < 0.0001), whereas *wenn* and *falls* did not differ significantly from each other in their ratings.

**TABLE 6:** Descriptive statistics of Experiment 4b.

Condition	Connective	EADV	Rating	*SE*
1	*falls*	Factive	2.04	0.07
2	*falls*	Non-factive	2.89	0.08
3	*weil*	Factive	3.61	0.08
4	*weil*	Non-factive	3.60	0.08
5	*wenn*	Factive	2.19	0.07
6	*wenn*	Non-factive	2.73	0.09

#### Discussion

Experiment 4b shows that both CCs are degraded with either type (i.e., factive/non-factive) of EADVs in comparison to the factive causal connective. Concerning CCs, it shows that sentences with either CC are degraded in co-occurrence with factive EADVs in comparison to those with non-factive EADVs. That is, the prediction that factive EADVs should favor *wenn* over *falls* was not borne out. This makes Contrast 5 as in the example of (12) invalid. We can thus conclude that in general, factive EADVs disprefer non-veridical contexts as created by both CCs, compared to non-factive EADVs, which are non-veridical as CCs.

These results have at least the following implications: First, in Karttunen’s (1971) term, EADVs are ‘semi-factive,’ i.e., they lose their factivity in certain contexts including questions, conditionals, and modals (see also Asher, 2000). But earlier work does not make a distinction among EADVs. Experiment 4b shows that EADVs indeed differ in terms of factivity, as argued in Liu (2012). Second, there exists a general constraint on co-occurring expressions with attitudinal meanings, namely, they need to agree with (or at least not clash with) each other. While this constraint needs further qualification and empirical validation, it is probably related to the notions of (in)coherence or (dis)harmony (Lyons, 1977). The factive causal connective is harmonious with both types of EADVs, as EADVs, despite their difference, express high degrees of commitment (i.e., toward full-commitment). Factive EADVs are less harmonious with CCs because the latter are non-factive, which are thus more coherent to combine with non-factive EADVs.

Furthermore, all ratings for sentences in Experiment 4b are very close to the midpoint of the scale, with the exception of sentences with *weil.* In other words, conditionals in general tend to be odd when antecedents are marked by EADVs. While this is an interesting result as far as the German factive and non-factive EADVs are concerned, it suggests that this might not be a useful manipulation for examining the differences between *wenn* and *falls.* If we compare Experiment 4a and 4b, there is one difference in the design in that in Experiment 4a, the two levels of the NPI factor were manipulated via the absence or the presence of NPIs, whereas the two levels of the EADV factor was manipulated via two different kinds of EADVs, not including a third level without EADVs. This difference is crucial in understanding the results: the *wenn/falls* contrast was significant in the NPI-absent conditions but not in the NPI-present conditions of Experiment 4a, whereas there was no difference between *wenn* and *falls* in Experiment 4b, which was potentially due to the lack of a cleaner comparison condition without EADVs.

## General Discussion and Conclusion

In this paper, I provided distributional properties of the two German CCs *wenn* and *falls* and argued that, while they are semantically both non-veridical, they differ in lexical pragmatics in that *falls* conveys a weaker speaker commitment toward the antecedent proposition than *wenn.*

In Experiment 1, subjects rated CCs in combination with events in the antecedent with varying degrees of likelihood by common knowledge. The results did not show any effect and thus were unable to confirm the proposed analysis. This might be due to the conversational nature of the meaning difference or due to the use of the binary scale which was not sensitive enough to measure subtle lexical pragmatics. Experiment 2 used the forced lexical choice task. It showed that *wenn* was preferred over *falls* in contexts where the protagonist had a high degree of credence in the antecedent proposition, and vice versa in contexts where the protagonist had a low degree of credence in the antecedent proposition. In Experiment 3, the conditional sentences were combined with a RC attached to the conditional antecedent, which conveyed the speaker’s high or low degree of credence in the antecedent proposition. Overall, it showed an interaction of RC and CC, with *wenn* being rated more natural than *falls* in the belief-condition, and vice versa in the disbelief-condition. This is in line with the proposed *wenn/falls* contrast and the results of Experiment 2. A closer look at the data revealed further differences due to the used RCs (*glauben/nicht glauben* vs. *un/wahrscheinlich*). With the RC containing *(nicht) glauben*, the experiment shows that the speaker can express positive or negative bias toward the antecedent proposition in the RCs, without causing incoherence with *wenn* or *falls.* This means that the lexical contrast between *wenn* and *falls* is part of the pragmatic (i.e., non-at-issue) rather than semantic or conventional meaning.

Experiment 4a (Liu, 2019), as summarized above, provides strong evidence for the proposed lexical pragmatic contrast between *wenn* and *falls.* Combining it with the results of Experiment 2, I argue that the proposed *wenn/falls* contrast is real. Experiment 4b tested connectives and EADVs in co-occurrence, and shows factive EADVs disprefer non-veridical CCs in comparison to non-factive EADVs. But it did not show a difference between the two CCs. In combination with the results of Experiments 1–3, this means that the *wenn/falls* contrast is subject to contextual modulations, i.e., it can be more visible in some and less so in others. In general, Experiment 4b also provides a first step toward understanding the interaction of co-occurring attitudinal expressions.

While CCs are argued to have no conditional meaning in the restrictor analysis, this paper shows that they can differ in meaning. The current study, in particular Experiment 2 and 3 in combination with the results of Liu (2019), provides evidence that the two frequently used German CCs *wenn* and *falls* differ in lexical pragmatics. The non-at-issue meanings of *wenn/falls* are reinforceable and cancellable, indicating their conversational nature and explaining the contextual effects found in the experiments. I relate their difference to the conveyed doxastic assumptions of the speaker, i.e., they express different degrees of speaker commitment. However, alternative analyses are possible.

One alternative is that the higher degree of speaker commitment in the case of *wenn* (in comparison to *falls*) may be due to its ambiguity between conditional and temporal interpretations (and the lack of ambiguity for *falls*), as temporal adverbial clauses are typically presupposed (Levinson, 1983, among others) and therefore factive. While we have seen examples with clearly conditional, non-temporal meaning, such as the biscuit-conditional in (4), the counterfactual conditional in (7), the conditionals with NPIs in (11) and (40), and the conditionals with EADVs in (12) and (41), we cannot rule out the possibility of the interference by the temporal reading of *wenn.* In fact, the results of Experiment 4a and 4b are in line with this possibility: in Experiment 4a, the *wenn/falls* contrast is significant without NPIs, that is, when the temporal interpretation is possible, whereas the difference is not visible with NPIs, i.e., when the temporal interpretation is not possible. In Experiment 4b, as the temporal reading was not possible across all the conditions, we were not able to detect any difference. However, I do not think this contradicts the current proposal for the *wenn/falls* contrast in terms of speaker commitment. In fact, Breindl et al. (2014, p. 265f) have argued that the choice of non-ambiguous *falls* leads to an implicature and that, in order to avoid the implicature, the speaker can consciously choose to use *wenn*, or vice versa, as we see in the examples of (28), possibly with the help of intonation: native speakers confirm that stressed *falls* strengthens the proposed WUI implicature. To sum up, I think the presence and absence of the temporal reading can be seen as a source (possibly out of several) for the *wenn/falls* contrast, with the implicature being the consequence in the choice. Here are two independent examples to illustrate the point.

With (13), we presented the difference between the necessity modal verb and the unmodalized variant in that the former triggers a weakened speaker commitment: _More committed_< unmodalized p, MUST p, POSSIBLY p>_Less committed_. It is unclear, however, how the difference arises. But the lexical choice of MUST can lead to the implicature linked to weakened speaker commitment, just as the choice between *falls* vs. *wenn.* Similarly, in (45), the speaker can use indicative mood or subjunctive I (Konjunktiv I) mood in the verb. Potts (2005, pp. 186, 187) argues that German Konjunctiv I is used to indicate the speaker’s wish “to distance himself from the propositional content expressed” or that “the speaker is not publicly committed to the truth of *p*,” but “It does not indicate that the speaker is committed to the negation of the propositional content in question.”


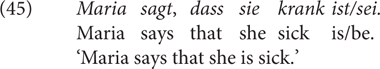


In other words, the choice between *ist/sei* indicates the degree to which the speaker intends to distance themselves from the given proposition. At the same time, the choice of *sei* over *ist* can give rise to an implicature, just as the choice of *falls* over *wenn* can. In this regard, it is also worth noting that Elder and Jaszczolt (2016) have put forward the notion of “remoteness from reality” in the context of conditionals. While their point is concerned with conditionals in general, e.g., *if* (with remoteness from reality, with regard to the antecedent) in comparison to *since/when* (without remoteness, i.e., alignment with reality), which they attribute to Grice (Elder and Jaszczolt, 2016: 41), the idea may nevertheless be relevant for understanding and modeling the *wenn/falls* contrast. The choice between them is then a choice between remoteness from or alignment with reality; the choice for *falls* over *wenn* can equally lead to an implicature of more focused or increased remoteness. These alternatives provide interesting perspectives that can help us to understand the mechanism behind lexical choices and implicatures, but I also think they are not incompatible with the current proposal.

It is to note, however, that this meaning difference is probably not the only aspect in which the two CCs differ. Consider (46): The sentence can have a conditional reading as in (46b), for which it is fine to replace *falls* with *wenn.* But it also has the reading in (46b), where *falls* can be best translated to “(just) in case” in English. The resulting sentence and its interpretation are different from canonical *falls*-conditionals and it is inappropriate to use *wenn*. Whether the contrast in terms of speaker commitment plays a role here will be left for future research.


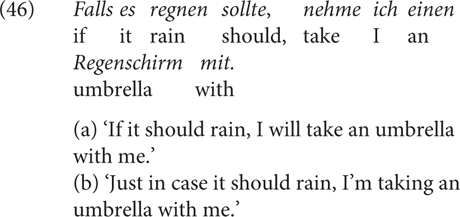


Secondly, *falls* does not always convey negative doxastic bias but sometimes it can convey negative bouletic bias. For example, in (47), the use of *falls* is compatible with the speaker’s dispreference but incompatible with their preference for the modified event.


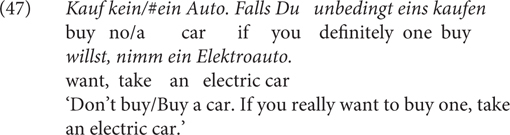


Methodologically speaking, this paper also shows that detection and validation of subtle differences in lexical pragmatics can be methodologically challenging. For example, the forced lexical choice study in Experiment 2 as well as the rating study using the speaker belief judgment task in Experiment 4a (Liu, 2019) show clear positive evidence for the contrast between *wenn/falls*. However, there was no evidence in the rating study in Experiment 1 and only weaker evidence in the rating study in Experiment 3. It is worth noting again that Experiments 1 and 3 used binary rating scales^19^, and Experiment 4b used a 5-point Likert scale, whereas Likert scales with less than 7 points are argued to be problematic (see, e.g., Liddell and Kruschke, 2018). Additionally, since the *wenn/falls* difference is of the conversational nature and supposed to be nuanced, a higher number of points (e.g., a 10-point scale) might be needed to make the scale sensitive enough to capture the difference, which I leave for future studies. A final note on the limitation of the current study is that the critical items of different numbers were used in combination with other experimental materials, which should be avoided in future to avoid potential confounds. I report experiments with or without evidence here to hopefully help future studies on testing lexical pragmatic differences.

In general, the results in the case study of *wenn/falls* also call for reconsiderations of Grice’s notion of ‘implicature’ from a probabilistic perspective, e.g., to model lexical pragmatics of near synonyms. We need a more gradable notion of implicature than the conventional and conversational distinction to model lexical semantics and pragmatics. Each case of near-synonyms has its own story in that the distance between them is gradient (cf. experimental evidence) rather than categorical. While this paper does not provide a general integrated theory for this purpose, it showcases the usefulness of speaker commitment scales as a formal tool for modeling lexical pragmatic contrast and the benefits of combining theoretical and experimental perspectives.

## Data Availability Statement

The raw data supporting the conclusions of this article will be made available by the authors, without undue reservation.

## Ethics Statement

The studies involving human participants were reviewed and approved by the Ethics Committee of Osnabrück University. The patients/participants provided their written informed consent to participate in this study.

## Author Contributions

ML conceived and conducted all the reported studies and wrote the manuscript.

## Conflict of Interest

The author declares that the research was conducted in the absence of any commercial or financial relationships that could be construed as a potential conflict of interest.

## Publisher’s Note

All claims expressed in this article are solely those of the authors and do not necessarily represent those of their affiliated organizations, or those of the publisher, the editors and the reviewers. Any product that may be evaluated in this article, or claim that may be made by its manufacturer, is not guaranteed or endorsed by the publisher.

## Footnotes

1 These expressions have different morphosyntactic properties (see Iatridou, 1991; Haegeman, 2003; Bhatt and Pancheva, 2006, a.o.). I put them under the unifying category of CC used in a semantic sense, due to the focus of the paper on the semantics and pragmatics of CCs and conditionals.

3 It is worth noting that subjunctive conditionals do not always presuppose or implicate the falsity of the antecedent proposition, and thus, are not always counterfactual (see Anderson, 1951; Iatridou, 2000; Starr, 2019). On the other hand, Arregui and Biezma (2016) argue that the counterfactuality implicature cannot be canceled without good reason.

4 The simple and complex CCs (used in purely descriptive terms) in natural language can differ in terms of among others, biconditionality, see Montoliìo (2000) on Spanish *a condición de* ‘on condition that’ and Liu and Barthel (2021) on German *nur wenn* ‘only if.’

5 Please note that the notion of commitment used in the paper is linked to the degree of the speaker’s belief or credence in a proposition, as conveyed by the modifying expressions. This is different from the notion used in, for example, Krifka (2015) or Geurts (2019) for modeling human communication.

6 The *Wortschatz* corpora (http://corpora.uni-leipzig.de, accessed October 27, 2020) of Leipzig University show that *wenn* has the frequency class 5, *falls* 10. The frequency class of a word FC(w) is calculated based on the occurrence frequency of the word “Fw” in comparison to the frequency of the most frequent word “Fmax,” in this case, *der* ‘the_MASC_,’ and is defined as FC(w) = [log[2](Fmax/Fw)]. The higher the frequency class, the rarer the word.

7 This does not mean that *wenn* is always ambiguous. For example, if we add a negative polarity item (NPI) such as *jemals* ‘ever’ into the antecedent, the resulting sentence (*wenn der Regen jemals aufhört* ‘if the rain ever stops’) only has a conditional reading. Furthermore, in this regard, the English *when* has also been argued to have both a temporal and a conditional reading, see Elder and Jaszczolt (2016) and their example (20) in it: *When you follow that through you’ve got the means to give rise to a change in the method.* Whether the ambiguity of the English *when* and the German *wenn* is comparable is an empirical question we will leave for future research.

8 It is to note that the speaker can use *falls* in order to not accommodate the antecedent proposition, for example, to indicate their doubt on A’s assertion. In comparison, (5) is supposed to show that in the case that the speaker does accommodate the antecedent proposition, they need to use *wenn* but not *falls.*

9 https://grammis.ids-mannheim.de/systematische-grammatik/2101 (accessed on 28.10.2020)

11 *Wenn* and *falls* can also be used in coordination, such as *wenn und falls, wenn oder falls* indicating that they have common semantic properties and distinctive features at the same time (Breindl et al., 2014).

15 As the fillers involved test materials of other experiments, they are not provided in the Supplementary Materials.

16 Please note that the studies reported in the paper were all conducted long before the Covid-19 Pandemic, as today (May 04, 2021 in Germany), it is not unusual that the shops are closed on Monday or Saturday.

17
https://www.soscisurvey.de/

18 All the studies reported were conducted between 2016 and 2017, and we only collected binary gender information. We collected age information using ranges for Experiments 1, 3, and 4b. While we also collected dialectal information (i.e., whether the subjects speak any German dialect and if yes, which ones) in the experiments, there was way too little data to report here. We did not control whether subjects were bilingual or not.

19 We used different scales in Experiments 1, 3, and 4 as they were conducted with different sub-experiments together.
